# Assessment of Patient Nondisclosures to Clinicians of Experiencing Imminent Threats

**DOI:** 10.1001/jamanetworkopen.2019.9277

**Published:** 2019-08-14

**Authors:** Andrea Gurmankin Levy, Aaron M. Scherer, Brian J. Zikmund-Fisher, Knoll Larkin, Geoffrey D. Barnes, Angela Fagerlin

**Affiliations:** 1 Department of Social Science, Middlesex Community College, Middletown, Connecticut; 2 Department of Internal Medicine, University of Iowa, Iowa City; 3 School of Public Health, University of Michigan, Ann Arbor; 4 Department of Oncology, Wayne State University School of Medicine, Detroit, Michigan; 5 University of Michigan Health System, Cardiovascular Medicine and Vascular Medicine, Ann Arbor; 6 Department of Population Health Sciences, University of Utah, Salt Lake City; 7 VA Salt Lake City Health Care System, Salt Lake City, Utah

## Abstract

**Importance:**

Patient disclosure to their clinician about experiencing an imminent threat is a critical step toward receiving support or assistance.

**Objective:**

To examine the frequency of patients not disclosing their experience of imminent threats to their clinician and their reasons for doing so.

**Design, Setting, and Participants:**

Survey study incorporating results from 2 national, nonprobability samples of 2011 US adults recruited from Amazon’s Mechanical Turk (MTurk) from March 16 to 30, 2015, and 2499 recruited from Survey Sampling International (SSI) from November 6 to 17, 2015. Data analysis was conducted from December 20 to 28, 2018.

**Main Outcomes and Measures:**

Self-reported nondisclosure of 4 types of imminent threats (depression, suicidality, abuse, and sexual assault) to their clinician and reasons for nondisclosure.

**Results:**

There were 2011 participants in the MTurk sample (1210 [60.3%] female; 1696 [60.2%] white; mean [SD] age, 35.7 [12.4] years; age range, 18-79 years) and 2499 participants (1273 [51.0%] female; 1968 [78.8%] white; mean [SD] age, 61.0 [7.6] years; age range, 50-91 years) in the SSI sample. Among those who reported experiencing at least 1 of the 4 imminent threats, 613 of 1292 MTurk participants (47.5%) and 581 of 1453 SSI participants (40.0%) withheld information from their clinician. The most commonly endorsed reasons for withholding this information included being embarrassed (MTurk: 72.7%; SSI: 70.9%), not wanting to be judged or lectured (MTurk: 66.4%; SSI: 53.4%), and not wanting to engage in a difficult follow-up behavior (MTurk: 62.4%; SSI: 51.1%). Respondents who experienced at least 1 of the 4 imminent threats had significantly higher odds of nondisclosure in both samples if they were female (MTurk: odds ratio [OR], 1.66 [95% CI, 1.30-2.11]; and SSI: OR, 1.33 [95% CI, 1.07-1.67]) or younger (MTurk: OR, 0.99 [95% CI, 0.98-1.00]; and SSI: OR, 0.98 [95% CI, 0.97-1.00]). Worse self-rated health was also associated with nondisclosure, but only in the SSI sample (OR, 0.85 [95% CI, 0.74-0.96]).

**Conclusions and Relevance:**

This study suggests that many people withhold information from their clinicians about imminent health threats that they face. A better understanding of how to increase patients’ comfort with reporting this information is critical to allowing clinicians to help patients mitigate these potentially life-threatening risks.

## Key Points

**Question:**

How common is it for patients to withhold information from clinicians about imminent threats that they face (depression, suicidality, abuse, or sexual assault), and what are common reasons for nondisclosure?

**Findings:**

This survey study, incorporating 2 national, nonprobability, online surveys of a total of 4510 US adults, found that at least one-quarter of participants who experienced each imminent threat reported withholding this information from their clinician. The most commonly endorsed reasons for nondisclosure included potential embarrassment, being judged, or difficult follow-up behavior.

**Meaning:**

These findings suggest that concerns about potential negative repercussions may lead many patients who experience imminent threats to avoid disclosing this information to their clinician.

## Teaser

This survey study assesses how often patients choose not to disclose to their clinicians imminent threats to their well-being (depression, suicidality, abuse, and sexual assault) and examines patient reasons for nondisclosure.

## Introduction

Gathering information from patients is essential to clinicians’ ability to help promote, maintain, and restore patients’ health. Previous research has demonstrated that patients frequently withhold relatively mundane, non–life-threatening information (eg, lack of exercise) from clinicians,^1,2^ but little is known about patient nondisclosure of information about experiencing an imminent threat to their lives (eg, being abused or suicidal). Prior research in this area has primarily been conducted outside of the United States and has a number of limitations: the study samples were constrained to a specific age range, sex, or geographic area; only 1 imminent threat was examined; and/or the scenarios were hypothetical.^3,4,5,6,7,8,9^ In this study, we examined the prevalence of US patients’ self-reported nondisclosure of information to their clinician about 4 imminent threats and their reasons for nondisclosure.

## Methods

The data for this study were collected as part of a larger survey study on patient nondisclosure to clinicians about medically relevant information, such as smoking, not understanding the clinician’s instructions, and not taking medication as prescribed.^1^ The study was initially conducted with a national, nonprobability sample of US adults aged 18 years and older recruited from Amazon’s Mechanical Turk (MTurk) from March 16 to 30, 2015. Owing to the generally younger skew of the MTurk sample, we recruited a second national, nonprobability sample of US adults aged 50 years and older from Survey Sampling International (SSI) from November 6 to 17, 2015. Sampling quotas were set with the SSI sample so that our sample matched the distribution of sex, age, and race/ethnicity in the US population for this age range. Additional methods for sampling and data collection are described elsewhere.^1^ Data analysis was conducted from December 20 to 28, 2018.

In this analysis, we focused on 4 imminent threats that patients did not disclose to clinicians; participants were asked whether they had ever avoided telling a clinician (defined in the survey as any medical caregiver, such as a doctor, physician’s assistant, or nurse) that they were depressed, had suicidal thoughts, had been abused, or had been sexually assaulted. Participants who did not indicate nondisclosure for a given threat were routed to a page asking whether they had experienced the threat before moving on to the next topic. Participants who indicated nondisclosure were routed to a page where they could select any of the reasons that were applicable for their nondisclosure before moving onto the next threat. The survey is included in the eAppendix in the Supplement.

The study was reviewed by the institutional review board of the University of Michigan Medical School. The MTurk survey was approved and the SSI survey was deemed exempt. Consent to participate was indicated electronically immediately prior to being routed to the webpage with the start of the survey. The study followed the American Association for Public Opinion Research (AAPOR) reporting guideline.

### Statistical Analysis

We recoded race and ethnicity into a dichotomous variable, with participants selecting white race/ethnicity being coded as 1 and participants not selecting white race/ethnicity being coded as 0. Health was reverse-coded so that higher values indicate better self-reported health.

Among the participants who experienced at least 1 of the threats, we report descriptive statistics, the percentage who reported ever having avoided telling a clinician about each of the threats, and the reasons they endorsed for doing so. We used multiple logistic regression to examine the demographic characteristics that were associated with whether or not the participant reported avoiding telling a clinician about any of the 4 imminent threats. To assess how often each reason for nondisclosure was endorsed, an aggregate measure for each reason was created by dividing the number of times a reason was endorsed by the total number of nondisclosures (eg, indicating “not wanting to be lectured or judged” for 2 of the 3 types of information a respondent reported not disclosing would yield a value of 66.7%).

A statistical significance level of 2-sided *P* < .05 was used. All analyses were conducted using SPSS statistical software version 22 (IBM) or Stata statistical software version Stata SE 14 (StataCorp).

## Results

### Participant Characteristics

Full demographic information for the full MTurk sample of 2011 participants (1210 [60.3%] female; 1696 [60.2%] white; mean [SD] age, 35.7 [12.4] years; age range, 18-79 years) and SSI sample of 2499 participants (1273 [51.0%] female; 1968 [78.8%] white; mean [SD] age, 61.0 [7.6] years; age range, 50-91 years) can be found in the eTable in the Supplement and in our earlier publication.^1^ In the MTurk sample, the 1292 respondents who experienced at least 1 of the 4 imminent threats had a mean (SD) age of 36 (12.1) years (range, 18-77 years) and were predominantly female (66.1%) and white (85.5%). In this sample, 45.4% had a bachelor’s degree or a higher degree, 80.5% rated their health as good to excellent, and 26.7% reported having a chronic illness. The 1453 respondents in the SSI sample who experienced at least 1 of the 4 imminent threats had a mean (SD) age of 60 (7.2) years (range, 50-91 years) and were predominantly female (59.4%) and white (78.3%). In this sample, 36.3% had a bachelor’s degree or a higher degree, 73.1% rated their health as good to excellent, and 45.5% reported having a chronic illness.

### Frequency of and Reasons for Patient Nondisclosure to Clinicians

**Figure 1.**
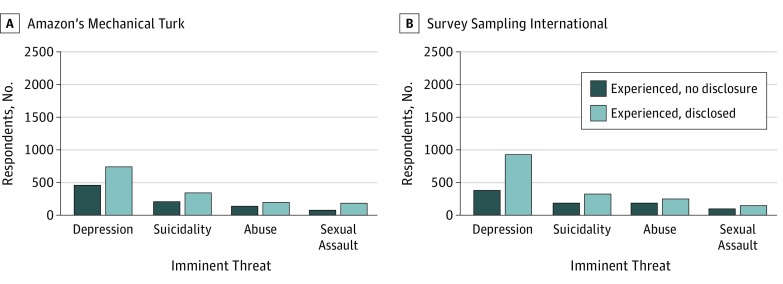
Nondisclosure and Disclosure Frequencies Among Respondents Who Experienced Each Imminent Threat Sample size was 2011 for Amazon’s Mechanical Turk (A) and 2499 for Survey Sampling International (B).

Of participants who reported experiencing at least 1 of the 4 threats (64.3% for MTurk and 58.1% for SSI), 613 of 1292 MTurk participants (47.5%) and 581 of 1453 SSI participants (40.0%) withheld at least 1 of the threats from their clinician. Figure 1 shows the frequencies of MTurk and SSI participants who reported having withheld information about each individual threat among those having experienced the individual corresponding threat. In the MTurk sample, abuse had the highest rate of nondisclosure (42.2%), followed by depression (38.1%), suicidality (37.8%), and sexual assault (28.8%). In the SSI sample, abuse had the highest rate of nondisclosure (42.3%), followed by sexual assault (41.6%), suicidality (37.0%), and depression (29.0%).

**Figure 2.**
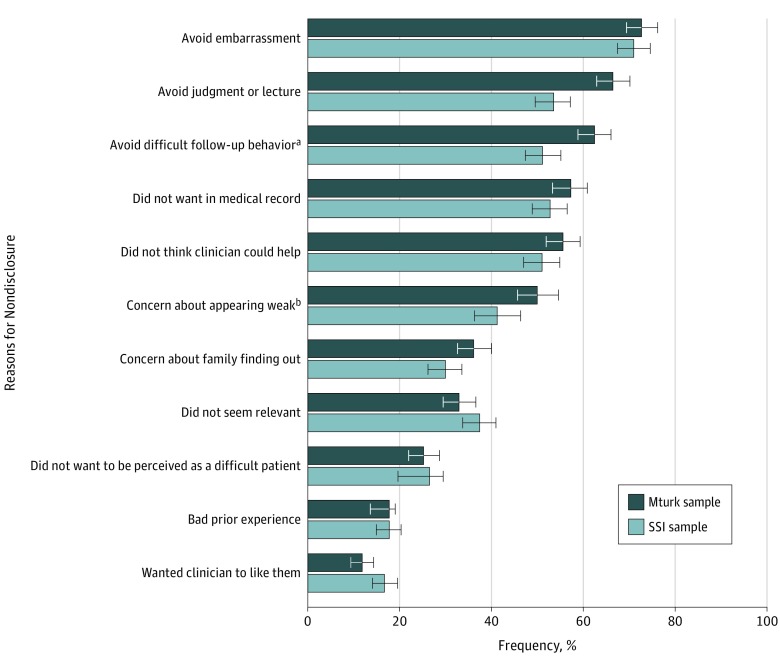
Percentage of Times a Reason Was Endorsed for Nondisclosure Across Types of Information Denominators were the total number of reasons for nondisclosure selected for each threat. Column totals may exceed 100% because participants could check multiple reasons. Error bars indicate 95% confidence intervals; MTurk, Amazon’s Mechanical Turk; and SSI, Survey Sampling International. ^a^Examples of potentially difficult behaviors were provided in this response option for each threat (eg, “take antidepressants or see a therapist” for suicidal thoughts). ^b^Reason only offered for nondisclosure of depression.

Among participants who withheld information, the most commonly indicated reasons included being embarrassed (MTurk: 72.7% [95% CI, 69.3%-76.0%]; and SSI: 70.9% [95% CI, 67.5%-74.4%]), not wanting to be judged or lectured (MTurk: 66.4% [95% CI, 62.8%-70.0%]; and SSI: 53.4% [95% CI, 49.5%-57.2%]), not wanting to have to engage in a difficult follow-up behavior (eg, “taking antidepressant medication or seeing a therapist” for suicidal thoughts) (MTurk: 62.4% [95% CI, 58.8%-66.0%]; SSI: 51.1% [95% CI, 47.2%-55.0%]), and not wanting the information in their medical record (MTurk: 57.1% [95% CI, 53.3%-60.8%]; and SSI: 52.7% [95% CI, 48.8%-56.5%]) (Figure 2).

### Risk Factors Associated With Nondisclosure to Clinicians

**Figure 3.**
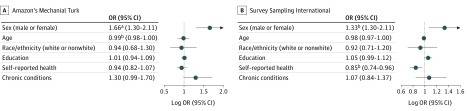
Odds of Nondisclosure of Imminent Threats The graphs show adjusted log odds ratios (ORs) for nondisclosure and the columns show ORs for nondisclosure in 1264 participants from the Amazon Mechanical Turk sample (A) and 1398 participants from the Survey Sampling International sample (B). ^a^*P* < .001. ^b^*P* < .05.

As shown in Figure 3, in both samples, those who were female (MTurk: odds ratio [OR], 1.66 [95% CI, 1.30-2.11]; and SSI: OR, 1.33 [95% CI, 1.07-1.67]) and younger (MTurk: OR, 0.99 [95% CI, 0.98-1.00]; and SSI: OR, 0.98 [95% CI, 0.97-1.00]) had significantly higher odds of nondisclosure of their experience to a clinician. Poorer self-reported health was also associated with significantly higher odds of nondisclosure in the SSI sample (OR, 0.85 [95% CI, 0.74-0.96]). Race/ethnicity and education were not significantly associated with nondisclosure in either sample.

## Discussion

Our findings indicate that clinicians do not receive information from a relatively high percentage of patients facing potentially life-threatening situations, which obviously precludes clinicians from helping patients to mitigate those risks. Women (who are significantly more likely to experience depression,^10^ suicide attempts,^11^ abuse,^12^ and sexual assault^12^) and those with poorer health (based on self-report) were significantly more likely to withhold this information from their clinician. Thus, consistent with our findings with less acute health risks (eg, lack of exercise),^1^ the patients who are in greatest need of assistance from their clinicians may be more likely to compromise their care by withholding critical information. The most commonly reported reasons for nondisclosure include concerns related to stigmatization (ie, being embarrassed, not wanting to be judged or lectured, not wanting the information in their medical record) and not wanting to have to engage in a difficult follow-up behavior.

### Limitations

This study has several limitations. A key limitation of our study is the use of online samples, which are not fully representative of the US population, but allowed us to obtain more demographically diverse samples than in-person convenience samples. The similarity in results between the 2 samples, despite their demographic differences, provides support for the validity of our findings. In addition, our study cannot speak to the degree of nondisclosure of these imminent threats (eg, whether a patient who is actively planning suicide said they are mildly suicidal or not suicidal at all) or the frequency of nondisclosure.

## Conclusions

This study reveals an important concern about clinician-patient communication: if patients commonly withhold information from clinicians about significant threats that they face, then clinicians are unable to identify and attempt to mitigate these threats. Thus, these results highlight the continued need to develop effective interventions that improve the trust and communication between patients and their clinicians, particularly for sensitive, potentially life-threatening topics.

## Notes

Supplement. **eAppendix.** Survey Questions **eTable.** Demographics of Full Samples
