# Supplementary material for: Assessment of Patient Nondisclosures to Clinicians of Experiencing Imminent Threats
Source: JAMA Netw Open. 2019 Aug 14;2(8):e199277. doi: 10.1001/jamanetworkopen.2019.9277 (PMC6694393; doi:10.1001/jamanetworkopen.2019.9277)
Supplement: Supplement. — eAppendix. Survey Questions eTable. Demographics of Full Samples [file jamanetwopen-2-e199277-s001.pdf]

Supplementary Online Content

Levy AG, Scherer AM, Zikmund-Fisher BJ, Larkin K, Barnes GD, Fagerlin A. Assessment of patient nondisclosures to clinicians of experiencing imminent threats. *JAMA Netw Open*. 2019;2(8):e199277. doi:10.1001/jamanetworkopen.2019.9277

eAppendix. Survey Questions
eTable. Demographics of Full Samples

This supplementary material has been provided by the authors to give readers additional information about their work.

© 2019 Levy AG et al. *JAMA Network Open.*

eAppendix: Survey questions

Introduction

Thank you for your time. This study is being conducted by the Center for Bioethics and Social Sciences in Medicine at the University of Michigan and the University of St. Joseph-Connecticut. By taking this survey, you can help researchers better understand how patients communicate with their physicians. Specifically, the information we learn will help physicians to better understand how to talk to their patients and help them feel comfortable. This survey will take about 10 minutes. Your name will not be recorded anywhere in this survey. All of your answers will be completely anonymous. If you choose to complete the survey, you can stop at any time. You may choose not to answer any questions you don't want to answer. If you want to stop and restart the survey at a later time, just click on the survey link in your email invitation again to continue where you left off. We will also collect some information about what operating system and browser you are using to view this survey and how long you spend on each page. This information is being collected for quality control. The Health Science and Behavioral Science Institutional Review Board (HSBS-IRB) has reviewed this study and has determined that it is exempt from IRB oversight. Your help means a lot to us. We thank you again for taking the time to complete this survey.

© 2019 Levy AG et al. *JAMA Network Open.*

On the following pages, we will ask you about types of situations that many patients avoid telling their health care providers about. By health care provider we mean any medical care giver such as a doctor, physician's assistant, or nurse. For each situation, we will ask you whether you have ever avoided telling a health care provider about it and if so, the reasons why you might have avoided sharing this information, such as: I was embarrassed, I wanted the health care provider to like me, I didn't want the information documented in my medical record. Please remember that this survey does not ask for your name or any other identifying information. Thank you again for your help.

Have you ever avoided telling a health care provider that you did not understand their instructions to you?

- ❍ Yes (1)
- ❍ No (2)

If Yes Is Selected, Then Skip To What were your reasons for not tellin...If No Is Selected, Then Skip To Have you ever not understood a health...

Have you ever not understood a health care provider's instructions?

- ❍ Yes (1)
- ❍ No (2)
- ❍ Wait- Now I remember! I have avoided telling a health care provider that I did not understand their directions to me. (3)

If No Is Selected, Then Skip To End of BlockIf Yes Is Selected, Then Skip To End of Block

You said that you have avoided telling a health care provider that you did not understand their directions to you. What were your reasons for not telling the health care provider this information? (Please click yes or no for each of the items below).

	No (1)	Yes (2)
I was embarrassed to admit that I didn't understand. (1)	❍	❍
I wanted the health care provider to like me. (2)	❍	❍
I didn't want the health care provider to think that I'm a difficult patient. (3)	❍	❍
I didn't want the health care provider to think that I'm stupid. (4)	❍	❍
I didn't want to take up any more of the health care provider's time. (5)	❍	❍
I didn't think it mattered. (6)	❍	❍
I had a bad experience with telling a health care provider that I didn't understand their instructions. (7)	❍	❍
Other (Please explain) (8)	❍	❍

Have you ever avoided telling a health care provider that you disagreed with his/her recommendation for you?

❍ Yes (1)

❍ No (2)

If Yes Is Selected, Then Skip To What were your reasons for not tellin...If No Is Selected, Then Skip To Have you ever disagreed with a health...

Have you ever disagreed with a health care provider's recommendation?

❍ Yes (1)

❍ No (2)

❍ Wait- Now I remember! I have avoided telling a health care provider that I disagree with his/her recommendation for me. (3)

If Yes Is Selected, Then Skip To End of BlockIf No Is Selected, Then Skip To End of Block

© 2019 Levy AG et al. *JAMA Network Open.*

You said that you have avoided telling a health care provider that you disagreed with his/her recommendation. What were your reasons for not telling the health care provider this information? (Please click yes or no for each of the items below).

	No (1)	Yes (2)
I was embarrassed to admit that I didn't agree. (1)	❍	❍
I wanted the health care provider to like me. (2)	❍	❍
I didn't want the health care provider to think that I'm a difficult patient. (3)	❍	❍
I didn't want the health care provider to think that I'm stupid. (4)	❍	❍
I didn't want to take up any more of the health care provider's time. (5)	❍	❍
I didn't think it mattered. (6)	❍	❍
I had a bad previous experience with telling a health care provider that I disagreed with him/her. (7)	❍	❍
Other (Please explain) (8)	❍	❍

Have you ever avoided telling a health care provider that you did not exercise or did not exercise regularly?

- ❍ Yes (1)
- ❍ No (2)

If Yes Is Selected, Then Skip To What were your reasons for not tellin...If No Is Selected, Then Skip To Have you ever disagreed with a health...

Have you ever not exercised regularly?

- ❍ Yes (1)
- ❍ No (2)
- ❍ Wait- Now I remember! I have avoided telling a health care provider that I did not exercise or did not exercise regularly. (3)

If Yes Is Selected, Then Skip To End of BlockIf No Is Selected, Then Skip To End of Block

© 2019 Levy AG et al. *JAMA Network Open.*

You said that you have avoided telling a health care provider that you did not exercise or did not exercise regularly. What were your reasons for not telling the health care provider this information? (Please click yes or no for each of the items below).

	No (1)	Yes (2)
I was embarrassed to admit that I did not exercise or exercise regularly. (1)	❍	❍
I wanted the health care provider to like me. (2)	❍	❍
I didn't want the health care provider to think that I'm a difficult patient. (3)	❍	❍
I didn't want the health care provider to think that I'm stupid. (4)	❍	❍
I didn't want to be judged or get a lecture about my behavior. (5)	❍	❍
I didn't want to hear about how bad inadequate exercise is for me. (6)	❍	❍
I didn't want to make a difficult change (i.e, exercising more) that the health care provider would then recommend. (7)	❍	❍
I didn't want this information in my medical record. (8)	❍	❍
I didn't want the health care provider to tell someone in my family. (9)	❍	❍
I didn't think it mattered. (10)	❍	❍
I had a bad previous experience with telling a health care provider this type of information. (11)	❍	❍
Other (Please explain) (12)	❍	❍

Have you ever avoided telling a health care provider that you had an unhealthy diet or how unhealthy your diet was?

❍ Yes (1)

❍ No (2)

If Yes Is Selected, Then Skip To What were your reasons for not tellin...If No Is Selected, Then Skip To Have you ever had an unhealthy diet?

© 2019 Levy AG et al. *JAMA Network Open.*

Have you ever had an unhealthy diet?

- ❍ Yes (1)
- ❍ No (2)
- ❍ Wait- Now I remember! I have avoided telling a health care provider that I had an unhealthy diet or how unhealthy my diet was. (3)

If No Is Selected, Then Skip To End of BlockIf Yes Is Selected, Then Skip To End of Block

© 2019 Levy AG et al. *JAMA Network Open.*

You said that you have avoided telling a health care provider that you had an unhealthy diet or how unhealthy your diet was. What were your reasons for not telling the health care provider this information? (Please click yes or no for each of the items below).

	No (1)	Yes (2)
I was embarrassed to admit that I had an unhealthy diet. (1)	❍	❍
I wanted the health care provider to like me. (2)	❍	❍
I didn't want the health care provider to think that I'm a difficult patient. (15)	❍	❍
I didn't want the health care provider to think that I'm stupid. (3)	❍	❍
I didn't want to be judged or get a lecture about my behavior. (4)	❍	❍
I didn't want to hear about how bad an unhealthy diet is for me. (5)	❍	❍
I didn't want to have to make a difficult change (i.e, improving my diet) that the health care provider would then recommend. (6)	❍	❍
I didn't want this information in my medical record. (7)	❍	❍
I didn't want the health care provider to tell someone in my family. (8)	❍	❍
I didn't think it mattered. (9)	❍	❍
I didn't think the health care provider could help me with this problem. (12)	❍	❍
I had a bad previous experience with telling a health care provider this type of information. (10)	❍	❍
Other (Please explain) (11)	❍	❍

© 2019 Levy AG et al. *JAMA Network Open.*

Have you ever avoided telling a health care provider that you took a certain medication (prescription, over-the-counter, or alternative medicines)? That is, have you ever deliberately NOT mentioned taking a certain medication?

- Yes (1)
- No (2)

If Yes Is Selected, Then Skip To What were your reasons for not tellin...If No Is Selected, Then Skip To Have you ever taken a prescription, o...

Have you ever taken a prescription, over-the-counter or alternative medicine that you thought a health care provider might be concerned about?

- Yes (1)
- No (2)
- Wait- Now I remember! I have avoided telling a health care provider that I took a certain medication (prescription, over-the-counter, or alternative medicines). (3)

If No Is Selected, Then Skip To End of BlockIf Yes Is Selected, Then Skip To End of Block

© 2019 Levy AG et al. *JAMA Network Open.*

You said that you have avoided telling a health care provider that you took a certain medication (prescription, over-the-counter, or alternative medicines). What were your reasons for not telling the health care provider this information? (Please click yes or no for each of the items below).

	No (1)	Yes (2)
I was embarrassed to admit that I took this medication. (1)	❍	❍
I wanted the health care provider to like me. (2)	❍	❍
I didn't want the health care provider to think that I'm a difficult patient. (15)	❍	❍
I didn't want the health care provider to think that I'm stupid. (3)	❍	❍
I didn't want to be judged or get a lecture about my behavior. (4)	❍	❍
I didn't want to hear about how bad taking this particular medication is for me. (5)	❍	❍
I didn't want to have to make a difficult change (i.e, stop taking this particular medication) that the health care provider would then recommend. (6)	❍	❍
I didn't want this information in my medical record. (7)	❍	❍
I didn't want the health care provider to tell someone in my family. (8)	❍	❍
I didn't think it mattered. (9)	❍	❍
I didn't think the health care provider could help me with this problem. (12)	❍	❍
I had a bad previous experience with telling a health care provider this type of information. (10)	❍	❍
Other (Please explain) (11)	❍	❍

© 2019 Levy AG et al. *JAMA Network Open.*

Have you ever avoided telling a health care provider that you did not take your prescription medications as instructed or how often you didn't take your prescription medications as instructed? (Note: this could mean taking your medication more or less often than instructed).
- ❍ Yes (1)
- ❍ No (2)

If Yes Is Selected, Then Skip To What were your reasons for not tellin...If No Is Selected, Then Skip To Have you ever not taken a prescriptio...

Have you ever not taken a prescription medication as instructed?
- ❍ Yes (1)
- ❍ No (2)
- ❍ Wait- Now I remember! I have avoided telling a health care provider that I did not take my prescription medication as instructed. (3)

If No Is Selected, Then Skip To End of BlockIf Yes Is Selected, Then Skip To End of Block

© 2019 Levy AG et al. *JAMA Network Open.*

You said that you have avoided telling a health care provider that you did not take your prescription medication as instructed. What were your reasons for not telling the health care provider this information? (Please click yes or no for each of the items below).

	No (1)	Yes (2)
I was embarrassed to admit that I did not take my perscription medication as instructed. (1)	❍	❍
I wanted the health care provider to like me. (2)	❍	❍
I didn't want the health care provider to think that I'm a difficult patient. (3)	❍	❍
I didn't want the health care provider to think that I'm stupid. (4)	❍	❍
I didn't want to be judged or get a lecture about my behavior. (5)	❍	❍
I didn't want to hear about how bad not taking my prescription medication as instructed is for me. (6)	❍	❍
I didn't want to have to make a difficult change (i.e, taking my medication as instructed) that the health care provider would then recommend. (7)	❍	❍
I didn't want this information in my medical record. (8)	❍	❍
I didn't want the health care provider to tell someone in my family. (9)	❍	❍
I didn't think it mattered. (10)	❍	❍
I didn't think the health care provider could help me with this problem. (13)	❍	❍
I had a bad previous experience with telling a health care provider this type of information. (11)	❍	❍
Other (Please explain) (12)	❍	❍

© 2019 Levy AG et al. *JAMA Network Open.*

Have you ever avoided telling a health care provider that you took SOMEONE ELSE'S prescription medications or how often you took SOMEONE ELSE'S prescription medications?

- ❍ Yes (1)
- ❍ No (2)

If Yes Is Selected, Then Skip To What were your reasons for not tellin...If No Is Selected, Then Skip To Have you ever taken someone else's pr...

Have you ever taken someone else's prescription medication?

- ❍ Yes (1)
- ❍ No (2)
- ❍ Wait- Now I remember! I have avoided telling a health care provider that I took someone else's prescription medication or how often I took someone else's prescription medication. (3)

If No Is Selected, Then Skip To End of BlockIf Yes Is Selected, Then Skip To End of Block

You said that you have avoided telling a health care provider that you took someone else's prescription medication or how often you took someone else's prescription medication. What

© 2019 Levy AG et al. *JAMA Network Open.*

were your reasons for not telling the health care provider this information? (Please click yes or no for each of the items below).

	No (1)	Yes (2)
I was embarrassed to admit that I took someone else’s prescription medication or how often I took someone else’s prescription medication. (1)	❍	❍
I wanted the health care provider to like me. (2)	❍	❍
I didn't want the health care provider to think that I'm a difficult patient. (15)	❍	❍
I didn't want the health care provider to think that I'm stupid. (3)	❍	❍
I didn't want to be judged or get a lecture about my behavior. (4)	❍	❍
I didn't want to hear about how bad taking someone else's prescription medication is for me. (5)	❍	❍
I didn't want to have to make a difficult change (i.e, quit taking someone else's prescription medication) that the health care provider would then recommend. (6)	❍	❍
I didn't want this information in my medical record. (7)	❍	❍
I didn't want the health care provider to tell someone in my family. (8)	❍	❍
I didn't think it mattered. (9)	❍	❍
I didn't think the health care provider could help me with this problem. (12)	❍	❍
I had a bad previous experience with telling a health care provider this type of information. (10)	❍	❍
Other (Please explain) (11)	❍	❍

© 2019 Levy AG et al. *JAMA Network Open.*

Have you ever avoided telling a health care provider that you drank alcohol when you shouldn't have or how much alcohol you drank?

- ❍ Yes (1)
- ❍ No (2)

If Yes Is Selected, Then Skip To What were your reasons for not tellin...If No Is Selected, Then Skip To Have you ever drank alcohol when you ...

Have you ever drank alcohol when you shouldn't have or drank more alcohol than you should have?

- ❍ Yes (1)
- ❍ No (2)
- ❍ Wait- Now I remember! I have avoided telling a health care provider that I drank alcohol when I shouldn't have or how much alcohol I drank. (3)

If No Is Selected, Then Skip To End of BlockIf Yes Is Selected, Then Skip To End of Block

© 2019 Levy AG et al. *JAMA Network Open.*

You said that you have avoided telling a health care provider that you drank alcohol when you shouldn't have or how much alcohol you drank. What were your reasons for not telling the health care provider this information? (Please click yes or no for each of the items below).

	No (1)	Yes (2)
I was embarrassed to admit that I drank alcohol when I shouldn’t have or how much alcohol I drank. (1)	❍	❍
I wanted the health care provider to like me. (2)	❍	❍
I didn't want the health care provider to think that I'm a difficult patient. (15)	❍	❍
I didn't want the health care provider to think that I'm stupid. (3)	❍	❍
I didn't want to be judged or get a lecture about my behavior. (4)	❍	❍
I didn't want to hear about how bad alcohol is for me. (5)	❍	❍
I didn't want to have to make a difficult change (i.e, quit drinking alcohol) that the health care provider would then recommend. (6)	❍	❍
I didn't want this information in my medical record. (7)	❍	❍
I didn't want the health care provider to tell someone in my family. (8)	❍	❍
I didn't think it mattered. (9)	❍	❍
I didn't think the health care provider could help me with this problem. (12)	❍	❍
I had a bad previous experience with telling a health care provider this type of information. (10)	❍	❍
Other (Please explain) (11)	❍	❍

© 2019 Levy AG et al. *JAMA Network Open.*

Have you ever avoided telling a health care provider that you smoked or how much you smoked?

- Yes (1)
- No (2)

If Yes Is Selected, Then Skip To What were your reasons for not tellin...If No Is Selected, Then Skip To Have you ever smoked?

Have you ever smoked?

- Yes (1)
- No (2)
- Wait- Now I remember! I have avoided telling a health care provider that I smoked or how much I smoked. (3)

If No Is Selected, Then Skip To End of BlockIf Yes Is Selected, Then Skip To End of Block

© 2019 Levy AG et al. *JAMA Network Open.*

You said that you have avoided telling a health care provider that you smoked or how much you smoked. What were your reasons for not telling the health care provider this information? (Please click yes or no for each of the items below).

	No (1)	Yes (2)
I was embarrassed to admit that I smoked or how much I smoked. (1)	❍	❍
I wanted the health care provider to like me. (2)	❍	❍
I didn't want the health care provider to think that I'm a difficult patient. (15)	❍	❍
I didn't want the health care provider to think that I'm stupid. (3)	❍	❍
I didn't want to be judged or get a lecture about my behavior. (4)	❍	❍
I didn't want to hear about how bad smoking is for me. (5)	❍	❍
I didn't want to have to make a difficult change (i.e, quit smoking) that the health care provider would then recommend. (6)	❍	❍
I didn't want this information in my medical record. (7)	❍	❍
I didn't want the health care provider to tell someone in my family. (8)	❍	❍
I didn't think it mattered. (9)	❍	❍
I didn't think the health care provider could help me with this problem. (12)	❍	❍
I had a bad previous experience with telling a health care provider this type of information. (10)	❍	❍
Other (Please explain) (11)	❍	❍

© 2019 Levy AG et al. *JAMA Network Open.*

Have you ever avoided telling a health care provider that you were depressed or how depressed you were?

- ❍ Yes (1)
- ❍ No (2)

If Yes Is Selected, Then Skip To What were your reasons for not tellin...If No Is Selected, Then Skip To Have you ever been depressed?

Have you ever been depressed?

- ❍ Yes (1)
- ❍ No (2)
- ❍ After giving it more thought, I do remember avoiding telling a health care provider that I was depressed or how depressed I was. (3)

If No Is Selected, Then Skip To End of BlockIf Yes Is Selected, Then Skip To End of Block

© 2019 Levy AG et al. *JAMA Network Open.*

You said that you have avoided telling a health care provider that you were depressed or how depressed you were. What were your reasons for not telling the health care provider this information? (Please click yes or no for each of the items below).

	No (1)	Yes (2)
I was embaressed to admit that I was depressed or how depressed I was. (1)	❍	❍
I wanted the health care provider to like me. (2)	❍	❍
I didn't want the health care provider to think that I'm a difficult patient. (12)	❍	❍
I didn't want the health care provider to think that I'm weak. (3)	❍	❍
I didn't want to be judged. (4)	❍	❍
I didn't want to have to make a difficult change (i.e, take an anti-depressant or see a therapist) that the health care provider would then recommend. (5)	❍	❍
I didn't want this information in my medical record. (6)	❍	❍
I didn't want the health care provider to tell someone in my family. (7)	❍	❍
I didn't think it mattered. (8)	❍	❍
I didn't think that the health care provider could help me with this problem. (9)	❍	❍
I had a bad previous experience with telling a health care provider this type of information. (10)	❍	❍
Other (Please explain) (11)	❍	❍

Have you ever avoided telling a health care provider that you had an embarrassing symptom or how severe that symptom was (such as rectal bleeding, genital warts, etc)?

❍ Yes (1)

❍ No (2)

If Yes Is Selected, Then Skip To What was the symptom/were the symptoms? If No Is Selected, Then Skip To Have you ever had an embarrassing sym...

© 2019 Levy AG et al. *JAMA Network Open.*

Have you ever had an embarrassing symptom?
- ❍ Yes (1)
- ❍ No (2)
- ❍ Wait- Now I remember! I have avoided telling a health care provider that I smoked or how much I smoked. (3)

If No Is Selected, Then Skip To End of BlockIf Yes Is Selected, Then Skip To End of Block

You said that you have avoided telling a health care provider that you had an embarrassing symptom or how severe the symptom was. What were your reasons for not telling the health care provider this information? (Please click yes or no for each of the items below).

	No (1)	Yes (2)
I was embarrassed by the symptom or how severe the symptom was. (1)	❍	❍
I wanted the health care provider to like me. (3)	❍	❍
I didn't want the health care provider to think that I'm a difficult patient. (14)	❍	❍
I didn't want to be judged. (4)	❍	❍
I was afraid to hear that the symptom meant something bad. (2)	❍	❍
I didn't want to have to make a difficult change (i.e, get an unpleasant test or treatment) that the health care provider would then recommend. (5)	❍	❍
I didn't want this information in my medical record. (6)	❍	❍
I didn't want the health care provider to tell someone in my family. (7)	❍	❍
I didn't think it mattered. (8)	❍	❍
I didn't think the health care provider could help me with this problem. (11)	❍	❍
I had a bad previous experience with telling a health care provider this type of information. (9)	❍	❍
Other (Please explain) (10)	❍	❍

© 2019 Levy AG et al. *JAMA Network Open.*

Have you ever avoided telling a health care provider that you had unsafe sex or how often you had unsafe sex?

- ❍ Yes (1)
- ❍ No (2)

If Yes Is Selected, Then Skip To What were your reasons for not tellin...If No Is Selected, Then Skip To Have you ever had unsafe sex?

Have you ever had unsafe sex?

- ❍ Yes (1)
- ❍ No (2)
- ❍ Wait- Now I remember! I have avoided telling a health care provider that I had unsafe sex or how often I had unsafe sex. (3)

If No Is Selected, Then Skip To End of BlockIf Yes Is Selected, Then Skip To End of Block

© 2019 Levy AG et al. *JAMA Network Open.*

You said that you have avoided telling a health care provider that you had unsafe sex or how often you had unsafe sex.   What were your reasons for not telling the health care provider this information? (Please click yes or no for each of the items below).

	No (1)	Yes (2)
I was embarrassed to admit that I had unsafe sex or how often I had unsafe sex. (1)	❍	❍
I wanted the health care provider to like me. (2)	❍	❍
I didn't want the health care provider to think that I'm a difficult patient. (15)	❍	❍
I didn't want the health care provider to think that I'm stupid. (3)	❍	❍
I didn't want to be judged or get a lecture about my behavior. (4)	❍	❍
I didn't want to hear how bad unsafe sex is for me. (5)	❍	❍
I didn't want to have to make a difficult change (i.e, stop having unsafe sex) that the health care provider would then recommend. (6)	❍	❍
I didn't want this information in my medical record. (7)	❍	❍
I didn't want the health care provider to tell someone in my family. (8)	❍	❍
I didn't think it mattered. (9)	❍	❍
I didn't think the health care provider could help me with this problem. (12)	❍	❍
I had a bad previous experience with telling a health care provider this type of information. (10)	❍	❍
Other (Please explain) (11)	❍	❍

© 2019 Levy AG et al. *JAMA Network Open.*

Have you ever avoided telling a health care provider that you used recreational drugs or how much you used recreational drugs?

- ❍ Yes (1)
- ❍ No (2)

If Yes Is Selected, Then Skip To What were your reasons for not tellin...If No Is Selected, Then Skip To Have you ever used recreational drugs?

Have you ever used recreational drugs?

- ❍ Yes (1)
- ❍ No (2)
- ❍ Wait- Now I remember! I have avoided telling a health care provider that I used recreational drugs how much I used recreational drugs. (3)

If No Is Selected, Then Skip To End of BlockIf Yes Is Selected, Then Skip To End of Block

© 2019 Levy AG et al. *JAMA Network Open.*

You said that you have avoided telling a health care provider that you used recreational drugs or how much you used recreational drugs. What were your reasons for not telling the health care provider this information? (Please click yes or no for each of the items below).

	No (1)	Yes (2)
I was embarrassed to admit that I had used recreational drugs or how much I had used recreational drugs. (1)	❍	❍
I wanted the health care provider to like me. (2)	❍	❍
I didn't want the health care provider to think that I'm a difficult patient. (15)	❍	❍
I didn't want the health care provider to think that I'm stupid. (3)	❍	❍
I didn't want to be judged or get a lecture about my behavior. (4)	❍	❍
I didn't want to hear how bad recreational drugs are for me. (5)	❍	❍
I didn't want to have to make a difficult change (i.e, stop using recreational drugs) that the health care provider would then recommend. (6)	❍	❍
I didn't want this information in my medical record. (7)	❍	❍
I didn't want the health care provider to tell someone in my family. (8)	❍	❍
I didn't think it mattered. (9)	❍	❍
I didn't think the health care provider could help me with this problem. (12)	❍	❍
I had a bad previous experience with telling a health care provider this type of information. (10)	❍	❍
Other (Please explain) (11)	❍	❍

© 2019 Levy AG et al. *JAMA Network Open.*

Have you ever avoided telling a health care provider that you have been abused or how severe the abuse was?

- Yes (1)
- No (2)

If Yes Is Selected, Then Skip To What were your reasons for not tellin...If No Is Selected, Then Skip To Have you ever used been abused?

Have you ever used been abused?

- Yes (1)
- No (2)
- After giving it more thought, I do remember avoiding telling a health care provider that I have been abused or how severe the abuse was. (3)

If No Is Selected, Then Skip To End of BlockIf Yes Is Selected, Then Skip To End of Block

© 2019 Levy AG et al. *JAMA Network Open.*

You said that you have avoided telling a health care provider that you have been abused or how severe the abuse was. What were your reasons for not telling the health care provider this information? (Please click yes or no for each of the items below).

	No (1)	Yes (2)
I was embarrassed to admit that I had been abused or how bad the abuse was. (1)	❍	❍
I wanted the health care provider to like me. (2)	❍	❍
I didn't want the health care provider to think that I'm a difficult patient. (11)	❍	❍
I didn't want to be judged. (3)	❍	❍
I didn't want to have to make a difficult change (i.e, leave the abuser or see a therapist) that the health care provider would then recommend. (4)	❍	❍
I didn't want this information in my medical record. (5)	❍	❍
I didn't want the health care provider to tell someone in my family. (6)	❍	❍
I didn't think it mattered. (7)	❍	❍
I didn't think the health care provider could help me with this problem. (8)	❍	❍
I had a bad previous experience with telling a health care provider this type of information. (9)	❍	❍
Other (Please explain) (10)	❍	❍

Have you ever avoided telling a health care provider that you have been sexually assaulted or raped?

❍ Yes (1)

❍ No (2)

If Yes Is Selected, Then Skip To What were your reasons for not tellin...If No Is Selected, Then Skip To Have you ever used been sexually assa...

© 2019 Levy AG et al. *JAMA Network Open.*

Have you ever been sexually assaulted or raped?

- ❍ Yes (1)
- ❍ No (2)
- ❍ After giving it more thought, I do remember avoiding telling a health care provider that I have been sexually assaulted or raped. (3)

If No Is Selected, Then Skip To End of BlockIf Yes Is Selected, Then Skip To End of Block

You said that you have avoided telling a health care provider that you have been sexually assaulted or raped. What were your reasons for not telling the health care provider this information? (Please click yes or no for each of the items below).

	No (1)	Yes (2)
I was embarrassed to admit that I had been sexually assaulted or raped. (1)	❍	❍
I wanted the health care provider to like me. (2)	❍	❍
I didn't want the health care provider to think that I'm a difficult patient. (11)	❍	❍
I didn't want to be judged. (3)	❍	❍
I didn't want to have to make a difficult change (i.e, see a therapist or file a police report) that the health care provider would then recommend. (4)	❍	❍
I didn't want this information in my medical record. (5)	❍	❍
I didn't want the health care provider to tell someone in my family. (6)	❍	❍
I didn't think it mattered. (7)	❍	❍
I didn't think the health care provider could help me with this problem. (8)	❍	❍
I had a bad previous experience with telling a health care provider this type of information. (9)	❍	❍
Other (Please explain) (10)	❍	❍

© 2019 Levy AG et al. *JAMA Network Open.*

Have you ever avoided telling a health care provider that you had suicidal thoughts or how often you had suicidal thoughts?

- ❍ Yes (1)
- ❍ No (2)

If Yes Is Selected, Then Skip To What were your reasons for not tellin...If No Is Selected, Then Skip To Have you ever had suicidal thoughts?

Have you ever had suicidal thoughts?

- ❍ Yes (1)
- ❍ No (2)
- ❍ After giving it more thought, I do remember avoiding telling a health care provider that I had suicidal thoughts or how often I had suicidal thoughts. (3)

If No Is Selected, Then Skip To End of BlockIf Yes Is Selected, Then Skip To End of Block

© 2019 Levy AG et al. *JAMA Network Open.*

You said that you have avoided telling a health care provider that you had suicidal thoughts or how often you had suicidal thoughts. What were your reasons for not telling the health care provider this information? (Please click yes or no for each of the items below).

	No (1)	Yes (2)
I was embarrassed to admit that I had suicidal thoughts or how often I had suicidal thoughts. (1)	❍	❍
I wanted the health care provider to like me. (2)	❍	❍
I didn't want the health care provider to think that I'm a difficult patient. (11)	❍	❍
I didn't want to be judged. (3)	❍	❍
I didn't want to have to make a difficult change (i.e, take an antidepressant or see a therapist) that the health care provider would then recommend. (4)	❍	❍
I didn't want this information in my medical record. (5)	❍	❍
I didn't want the health care provider to tell someone in my family. (6)	❍	❍
I didn't think it mattered. (7)	❍	❍
I didn't think the health care provider could help me with this problem. (8)	❍	❍
I had a bad previous experience with telling a health care provider this type of information. (9)	❍	❍
Other (Please explain) (10)	❍	❍

What is your gender?
- ❍ Male (1)
- ❍ Female (2)
- ❍ Other/Transgender (3)

What is your age?

© 2019 Levy AG et al. *JAMA Network Open.*

Are you Hispanic or Latino/a?
- ❍ Yes (1)
- ❍ No (2)

What is your race? (Select all that apply)
- ❑ White or Caucasian (1)
- ❑ Black or African-American (2)
- ❑ American Indian or Alaska Native (3)
- ❑ Asian or Asian-American (4)
- ❑ Pacific Islander or Native Hawaiian (5)
- ❑ Other (please specify) (6) ____________________

What is the highest level of education you have completed?
- ❍ None (1)
- ❍ Elementary school (2)
- ❍ Some high school, but no diploma (3)
- ❍ High school (Diploma or GED) (4)
- ❍ Trade school (5)
- ❍ Some college, but no degree (6)
- ❍ Associate's degree (AA, AS, etc.) (7)
- ❍ Bachelor's degree (BS, BA, etc.) (8)
- ❍ Master's degree (MA, MPH, etc.) (9)
- ❍ Doctoral/Professional degree (PhD, MD, etc.) (10)

In general, would you say your health is:
- ❍ Excellent (1)
- ❍ Very good (2)
- ❍ Good (3)
- ❍ Fair (4)
- ❍ Poor (5)

Do you have a chronic illness (e.g., diabetes, heart disease, asthma, etc)?
- ❍ Yes (1)
- ❍ No (2)

Thank you for your participation in this study. If you have any questions about this survey please email xxxxxxx.

© 2019 Levy AG et al. *JAMA Network Open.*

eTable: Demographics of full samples

	**MTurk (N=2011)**	**SSI (N=2499)**
**Age** mean (SD)	35.7 (12.4)	61.0 (7.59)
**Gender**		
Female	1210 (60.3%)	1273 (51.0%)
Male	782 (39.0%)	1218 (48.9%)
Transgender or other gender minority	13 (0.7%)	2 (0.1%)
**Race**[a]		
White or Caucasian	1696 (60.2%)	1968 (78.8%)
Black or African-American	175 (8.7%)	363 (14.5%)
American Indian or Alaska Native	46 (2.3%)	41 (1.6%)
Asian or Asian-American	117 (5.8%)	103 (4.1%)
Pacific Islander or Native Hawaiian	8 (0.4%)	6 (0.2%)
Other racial minority	35 (1.7%)	61 (2.4%)
**Education**		
High school (diploma or GED) or less	211 (10.5%)	476 (19.2%)
Some college or 2-year degree	821 (41.0%)	985 (39.6%)
4-year degree or more	974 (48.5%)	1023 (41.2%)
**Self-reported health**		
Excellent	242 (12.1%)	233 (9.4%)
Very good	744 (37.1%)	834 (33.6%)
Good	720 (35.9%)	905 (36.5%)
Fair	261 (13.0%)	430 (17.4%)
Poor	39 (1.9%)	78 (3.2%)
**Chronic illness diagnosis**		
No	1553 (77.5%)	1504 (60.8%)
Yes	450 (22.5%)	969 (39.2%)

Notes: Values indicate number of participants (percentage of total sample). [a]Participants could select more than one race, so values may add up to more than 100%.

© 2019 Levy AG et al. *JAMA Network Open.*
